# Fluoroscopy-Guided Blockade of the Greater Occipital Nerve in Cadavers: A Comparison of Spread and Nerve Involvement for Different Injectate Volumes

**DOI:** 10.1155/2020/8925895

**Published:** 2020-09-22

**Authors:** Zhanfeng Song, Shuming Zhao, Jianqing Ma, Zhanyong Wu, Sidong Yang

**Affiliations:** ^1^Department of Orthopaedic Surgery, Orthopaedic Hospital of Xingtai, Xingtai 054000, China; ^2^Xingtai Institute of Orthopaedics, Xingtai 054000, China; ^3^Department of Orthopaedic Surgery, The Third Hospital of Hebei Medical University, Shijiazhuang 050051, China

## Abstract

**Background:**

Fluoroscopy-guided blockade of the greater occipital nerve (GON) is an accepted method for treating the symptoms of cervicogenic headaches (CGHs). However, the spread patterns among different injectate volumes of fluoroscopy-guided GON blocks are not well defined.

**Objective:**

A cadaveric study was established to determine the spread patterns of different volumes of dye injectate within a fluoroscopic GON block. *Study Design*. Cadaveric study. *Setting*. Xingtai Institute of Orthopaedics; Orthopaedic Hospital of Xingtai.

**Methods:**

15 formalin-fixed cadavers with intact cervical spines were randomized in a 1 : 1 : 1 ratio to receive a fluoroscopy-guided GON injection of a 2, 3.5, or 5 ml volume of methylene blue. The suboccipital regions were dissected to investigate nerve involvement.

**Results:**

The suboccipital triangle regions, including the suboccipital nerves and GONs, were deeply stained in all cadavers. The third occipital nerve (TON) was stained in 7 of 10 administered 2 ml injections and in all the 3.5 ml and 5 ml injections. Compared to the 3 ml injectate group, the 5 mL cohort consistently saw injectate spreading to both superficial and distant muscles. *Limitations*. Given that cadavers were used in this study, cadaveric soft tissue composition and architecture can potentially become distorted and consequently affect injectate diffusion.

**Conclusions:**

A 3.5 or 5 mL fluoroscopy-guided GON injection of methylene blue successfully stains the GON, TON, and suboccipital nerves. This suggests that such an injection would generate blockade of all three nerve groups, which may contribute to the efficacy of the block for CGH. A volume of 3.5 ml may be enough for the performance of a fluoroscopy-guided GON block for therapeutic purposes.

## 1. Introduction

A cervicogenic headache (CGH) is a well-recognised headache syndrome. It has been estimated that 1.0–4.1% of the population experience CGHs [1–3]. There are multiple therapeutic modalities for managing CGHs, including pharmacological management, interventional therapies, and surgical interventions. CGHs remain a major problem, as most therapeutic regimens are still followed by the recurrence of pain. To date, arguably, the most effective treatment for CGH is the interruption of pain transmission via the occipital nerves or their component nerve roots or ganglia, either by means of an anaesthetic block or through a surgical intervention [4].

Greater occipital nerve (GON) blockade procedures are commonly performed for CGHs, which have a dual role in both supporting a diagnosis and providing pain relief. As a result of a localised and painful pressure point at the greater occipital nerve, a confined injection of corticosteroid and local anaesthetics is classified as 1B + for the management of CGH when conservative treatments fail [5]. The suboccipital compartment injection was introduced in 1980 by Racz et al. [6] and has been subsequently recommended by others. The fluoroscopy-guided GON block technique is an accepted treatment method for the symptoms of CGHs and is superior to the classical GON technique [7].

There is a lack of consensus on the optimal injectate volume for successful GON block treatment. Baek et al. found 5 ml to be the suitable volume for ultrasound-guided GON block, but this work has not been replicated to fluoroscopy-guided GON block [8]. In light of spread patterns between different injectate volumes in fluoroscopy-guided GON blocks not being clear, a cadaveric study was designed to determine the spreading of different volumes of dye injectate in a fluoroscopic GON block.

## 2. Methods

A request for exemption was approved by our institutional review board for dissection (no. ZCKT-0018). Injections using the fluoroscopy-guided GON block technique were performed on 15 formalin-fixed cadavers with intact cervical spines. The cadavers were randomized in a 1 : 1 : 1 ratio to receive injection of a 2, 3.5, or 5 ml volume of methylene blue (1% methylthioninium chloride injection, Hubei Jumpcan Pharmaceutical Co., Ltd., Taixing, China). The cadavers were placed in a prone position with the neck slightly flexed. 2 to 3 cm lateral to the occipital protuberance, at the nuchal ridge, the skin entry point was marked. The needle was advanced in a posteroanterior direction toward the arch of C1. Once the needle had been advanced 2 to 3 cm into the tissue, the needle was further advanced under a lateral fluoroscopic view toward the arch of C1. The needle advance was ceased 1 to 2 mm posterior to the C2 spinous process, posterior and inferior to the C1 arch. After needle placement was assessed and validated in both the anterior-posterior and lateral views, 2, 3.5, or 5 ml of methylene blue was injected accordingly. Postinjection cadaveric dissections were performed by an independent anatomical specialist (who was not involved in block performance) to evaluate the location, vertical spread, and grossly observed injectate coating of the nerves. The dissection process was commenced by reflecting the skin of the occipital and posterior neck regions to reveal the subcutaneous tissue. The trapezius muscle and splenius capitis were carefully dissected along its fascial plane to the level of the semispinalis capitis muscle. Dissections were done to evaluate injectate spreading by cutting the semispinalis capitis from its occipital attachment and along its anterior fascia, reflected laterally to identify the GON and third occipital nerve (TON).

## 3. Results

**Figure 1:**
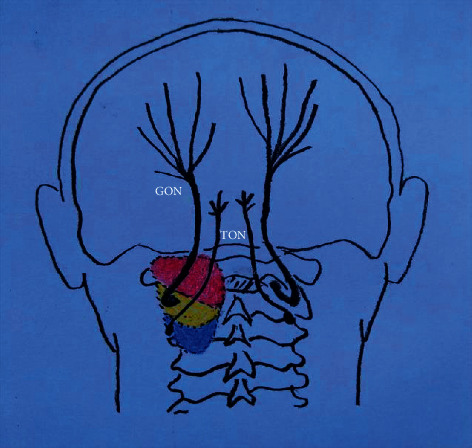
A schematic diagram demonstrating the extent of dye spread between different injectate volumes. The red area indicates the distribution of 2 ml injectate volume. The red and yellow areas denote the distribution of 3.5 ml injectate volume. The red, yellow, and blue areas show the distribution of 5 ml injectate volume. GON: greater occipital nerve; TON: third occipital nerve.

**Figure 2:**
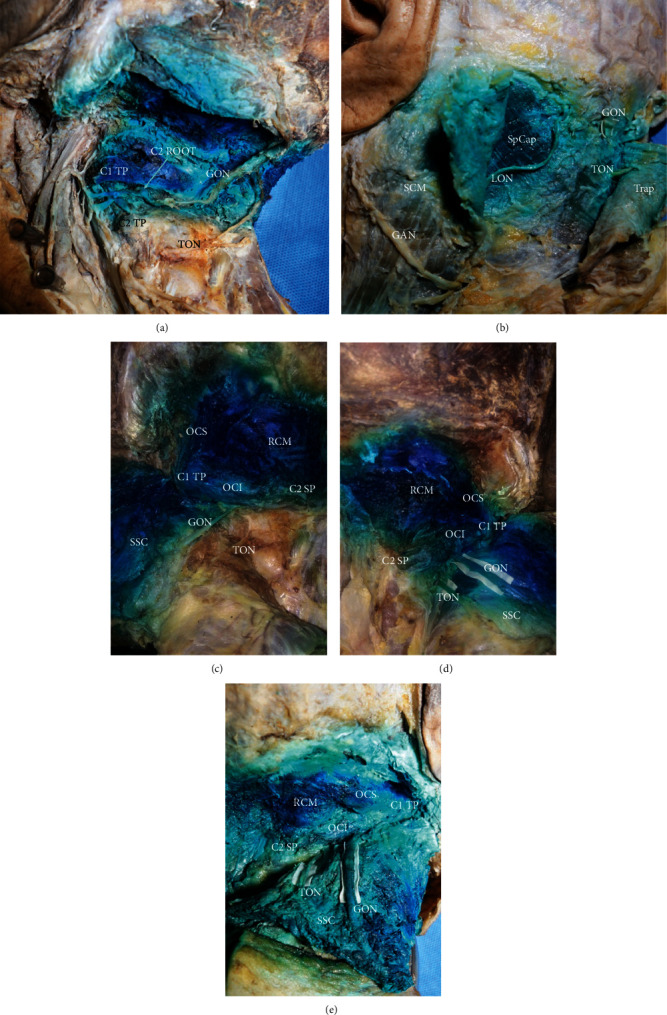
The images of the injectate distribution. (a) Test cadaver post left-sided injection with 2 ml of injectate. The dissection revealed coating of the second cervical nerve root. (b) Test cadaver post left-sided injection with 5 ml of injectate. Dissection revealed coating of the mastoid tip of sternocleidomastoid. (c) Test cadaver post left-sided injection with 2 ml of injectate. The dissection revealed coating of GON without coating of TON. (d) Test cadaver post right-sided injection with 3.5 ml of injectate. The dissection revealed coating of both GON and TON. (e) Test cadaver post right-sided injection with 5 ml of injectate. The dissection revealed coating of both GON and TON. GON: greater occipital nerve; TON: third occipital nerve; LON: lesser occipital nerve; GAN: great auricular nerve; OCI: obliquus capitis inferior; OCS: obliquus capitis superior; RCM: rectus capitis major; SSC: semispinalis capitis; SCM: sternocleidomastoid; SpCap: splenius capitis; Trap: trapezius; TP: transverse process; SP: spinous process.

In total, 15 cadavers were included in the study: nine men and six women with an average age of 73.6 years and an average weight of 55.8 kg. Ten injections were performed in each group using a 2, 3.5, or 5 ml volume, respectively. Thirty suboccipital triangles were dissected, and the distribution of dye in relation to the GON and TON was observed. In the 2 ml injectate group, most of the dye was contained within the suboccipital triangle (Figure 1). The obliquus capitis inferior, obliquus capitis superior, rectus capitis major, rectus capitis minor, and the deep part of the semispinalis capitis were deeply stained. Five longissimus capitis and one splenius capitis were partly coloured by the dye. All suboccipital nerves (which innervates the obliquus capitis inferior, obliquus capitis superior, rectus capitis major, and semispinalis capitis muscles) and GONs were deeply stained, as well (Table 1). All second cervical nerve roots were reached by the dye (Figure 2(a)). Seven of ten TONs were stained (Figure 2(c)). In the 3.5 ml injectate group, except for the coloured muscles and nerves in the 2 ml injectate cohort, the dye continued to spread within the semispinalis capitis, and caudad spread to the C3 level (Figure 1). All TONs were stained (Figure 2(d)). All longissimus capitis, splenius capitis, and sternocleidomastoid were partly stained. Two trapeziuses were partly coloured by the dye (Table 1). In the 5 ml injectate group, except for the coloured muscles and nerves in the 3.5 ml cohort, full-thickness semispinalis capitis was stained, and the dye caudad spread to the C4 level (Figure 1). All TONs were reached by the dye (Figure 2(e)); all longissimus capitis, splenius capitis, and sternocleidomastoid were partly stained. Six trapeziuses were partly coloured by the dye (Table 1 and Figure 2(b)).

## 4. Discussion

This study is the first to report staining of GON, TON, and suboccipital nerves from fluoroscopy-guided GON injections. In the 2 ml injectate groups, 70% of TONs and 100% of both GONs and suboccipital nerves were simultaneously stained. After increasing the injectate volume to 3.5 or 5 ml, all the GON, TON, and suboccipital nerves were deeply stained. CGHs are caused by upper cervical spine disorders of anatomical structures innervated by the first three cervical spinal nerves. The rationale for using a GON block as a treatment for CGHs comes from the proximity of sensory neurons in the upper three cervical spinal cords to the trigeminal nucleus caudalis (TNC) neurons and the convergence of sensory input to TNC neurons from both the cervical and trigeminal fibres [9, 10]. Therefore, all structures innervated by the first three cervical spinal nerves are possible sources for CGHs [11]. A previous anatomical study demonstrated that when the interconnections between C1–C2–C3 (54–65.4%) can be observed, the interconnections are then often known to be associated with failed surgical treatment or incomplete success of pain interventions [12]. These findings suggest that CGH might be more efficiently relieved by blocking both the suboccipital muscles and the dorsal rami of C1, C2, and C3 simultaneously. In this study, most of the dorsal rami of the first three cervical spinal nerves in all three groups were stained. The staining observed in our study supports the efficacy of the fluoroscopy-guided GON block given the known pathophysiology of CGH.

The findings from this study may strengthen the evidence for the anatomic basis of the extended duration observed clinically with fluoroscopy-guided GON blocks. Lauretti GR et al. reported that the classical technique resulted in 2 weeks of analgesia and the fluoroscopy-guided GON block resulted in at least 24 weeks of analgesia, probably secondary to central desensitization. They speculated that the subcompartmental administration of the drug would be close enough to permit spread to nearby dorsal ganglions and contribute to modulate central sensitization [7]. Some studies have reported the role of the dorsal root ganglia (DRG) in peripheral and central sensitization [13, 14]. In the present study, the results provide evidence of dyeing of all C2 roots (including DRGs), which could provide an important anatomical basis for the therapeutic mechanism of modulating peripheral and central sensitization in a fluoroscopic GON block.

Voigt et al. reviewed the literature on the usefulness of the occipital nerve block and found there were no clear consensus volumes for injection. Previous studies evaluated volumes between 0.5 and 10 mL injected at a single site, with the most common volume being 3 ml [15]. Compared to either 10 or 15 mL injectate volumes, Lauretti et al. indicated that a 5 ml volume of injectate for fluoroscopy-guided GON block was sufficient to result in efficient analgesia for CGH [7]. Baek et al. compared the injectate spread and nerve involvement between 1 ml volume and 5 ml volume using ultrasound-guided GON blocks at the C2 level and found 5 mL volume of injectate seemed to be suitable for therapeutic purpose [8]. According to our experience in clinical practice, we often used a 3 to 5 ml volume for fluoroscopy-guided GON injection and achieved satisfactory results. In the present study, although 3.5− or 5 mL fluoroscopy-guided GON injection of methylene blue stains the GON, TON, and suboccipital nerves, the 5 ml cohort spread dorsally to superficial and distant muscles, whereas the 3.5 ml injectate cohort was mainly contained to the dorsal rami of the first three cervical spinal nerves without extensive extravasation. Thus, compared to the 5 ml injectate volume, a volume of 3.5 ml seemed to be enough for the performance of a fluoroscopy-guided GON block for therapeutic purpose.

## 5. Limitations

Considering that we used cadavers, it is important to note that cadaveric soft tissue composition and architecture may become distorted and affect the injectate diffusion. The movement of neck and absorption of drug in living subjects can also affect the injectate diffusion. Hence, the injectate diffusion in living subjects could not be completely in accord with cadaver findings. Though the cadaveric design presents limitations when translating cadaveric findings to a clinical setting, the current results could provide some crucial parameters for clinical practice in terms of the consistency of spread within the different subgroups, and it still needs to be further studied.

## 6. Conclusion

A 3.5 or 5 mL fluoroscopy-guided GON injection of methylene blue stains the GON, TON, and suboccipital nerves. This suggests that such an injection would generate blockade of all three nerve groups, which may contribute to the efficacy of the block for CGH. A volume of 3.5 ml may be enough for the performance of a fluoroscopy-guided GON block for therapeutic purpose.

## Figures and Tables

**Table 1:** Comparison of dye staining between suboccipital compartment block with 2, 3.5, and 5 ml of methylene blue.

Anatomical structures	2 ml dye (*n* = 10)	3.5 ml dye (*n* = 10)	5 ml dye (*n* = 10)
*Nerves*			
Suboccipital nerve	10 (100%)	10 (100%)	10 (100%)
GON	10 (100%)	10 (100%)	10 (100%)
Third occipital nerve	7 (70%)	10 (100%)	10 (100%)
LON	0	10 (100%)	10 (100%)

*Muscles*			
Obliquus capitis inferior	10 (100%)	10 (100%)	10 (100%)
Rectus capitis major	10 (100%)	10 (100%)	10 (100%)
Rectus capitis minor	10 (100%)	10 (100%)	10 (100%)
Obliquus capitis superior	10 (100%)	10 (100%)	10 (100%)
Semispinalis capitis	10 (100%)	10 (100%)	10 (100%)
Longissimus capitis	5 (50%)	10 (100%)	10 (100%)
Splenius capitis	1 (10%)	10 (100%)	10 (100%)
Sternocleidomastoid (mastoid tip)	0	10 (100%)	10 (100%)
Trapezius	0	2 (20%)	6 (60%)

## Data Availability

The raw data used to support the findings of this study are included within the article.
